# Integrated transcriptome and metabolome analysis revealed that HaMYB1 modulates anthocyanin accumulation to deepen sunflower flower color

**DOI:** 10.1007/s00299-023-03098-3

**Published:** 2024-02-21

**Authors:** Siqi Ma, Hanlin Zhou, Tingting Ren, Er-ru Yu, Bin Feng, Juying Wang, Chengsheng Zhang, Chao Zhou, Yiqiang Li

**Affiliations:** 1 Marine Agriculture Research Center/Key Laboratory of Synthetic Biology of Ministry of Agriculture and Rural Affairs, Tobacco Research Institute of Chinese Academy of Agricultural Sciences, Qingdao, 266101 China; 2 Yichang Key Laboratory of Omics-Based Breeding for Chinese Medicines, Key Laboratory of Three Gorges Regional Plant Genetics and Germplasm Enhancement/Biotechnology Research Center, China Three Gorges University, Yichang, 443002 China; 3 Guizhou Institute of Oil Crops, Guizhou Academy of Agricultural Science, Guiyang, 550006 China; 4 Technical Innovation Center for Comprehensive Utilization of Saline-Alkali Land in Huangsanjiao Agricultural High-Tech, Dongying, 257000 China

**Keywords:** Sunflower, Anthocyanins, MYB transcriptional factors

## Abstract

**Key message:**

HanMYB1 was found to play positive roles in the modulation of anthocyanins metabolism based on the integrative analysis of different color cultivars and the related molecular genetic analyses.

**Abstract:**

As a high value ornamental and edible crop with various colors, sunflowers (*Helianthus annuus* L.) provide an ideal system to understand the formation of flower color. Anthocyanins are major pigments in higher plants, which is associated with development of flower colors and ability of oxidation resistance. Here, we performed an integrative analysis of the transcriptome and flavonoid metabolome in five sunflower cultivars with different flower colors. According to differentially expressed genes and differentially accumulated flavonoids, these cultivars could be grouped into yellow and red. The results showed that more anthocyanins were accumulated in the red group flowers, especially the chrysanthemin. Some anthocyanins biosynthesis-related genes like UFGT (UDP-glycose flavonoid glycosyltransferase) also expressed more in the red group flowers. A MYB transcriptional factor, HanMYB1, was found to play vital positive roles in the modulation of anthocyanins metabolism by the integrative analysis. Overexpressed HanMYB1 in tobacco could deepen the flower color, increase the accumulation of anthocyanins and directly active the express of *UFGT* genes. Our findings indicated that the MYB transcriptional factors provide new insight into the dynamic regulation of the anthocyanin biosynthesis in facilitating sunflower color formation and anthocyanin accumulation.

**Supplementary Information:**

The online version contains supplementary material available at 10.1007/s00299-023-03098-3.

## Introduction

Sunflower (*Helianthus annuus* L.) is a crop with high ornamental value due colorful flowers, and a popular crop in floriculture because of its agronomically desirable characteristics, such as short growth cycle and resistance to abiotic stress (Kumari and Bhatla 2021). Flavonoids are the major pigment in higher plants, in addition to carotenoids and betalains (Polturak et al. 2018). One of the major flavonoids in flower is anthocyanins. Anthocyanins express colors ranging from orange, purple, blue, and even black colors(Hashimoto et al. 2011). Flavonoids are also responsible for different flower colors, including yellow, purple, and so on (Iwashina 2015). More than 8000 types of flavonoids have been reported and they are widely exist in flowering plants (Tanaka et al. 2010). Flavonoids not only influence flower and fruit color, but also participate in flavor development, plant-environment interactions (Koes et al. 2005; Ma et al. 2014, 2020; Agati and Tattini 2010).

Phenylalanine is a precursor in flavonoid biosynthesis pathways, and the flavonoids are accumlated through multi-step processes (Zhao et al. 2020). The processes begin with the conversion of phenylalanine into coumaroyl CoA, by sequential actions of three enzymes, i.e., phenylalanine ammonia lyase (Lucheta et al.), cinnamate 4-hydroxylase, and 4-coumarate CoA ligase (Fowler and Koffas 2009). Subsequently, coumaroyl CoA combines with malonyl CoA to form chalcone, the reaction is catalyzed by chalcone synthase (Vidal-Melgosa et al. 2021). Under the catalyzing by chalcone isomerase (CHI), chalcone transforms to naringenin. Then naringenin could be further transformed into different classes of flavonoids (Wan et al. 2020; Koes et al. 2005). Flavanones (e.g., hesperetin, eriodictyol, and sakuranetin) can be converted by naringenin through the hydroxylation and methylation. Flavone (e.g., apigenin, luteolin, and diosmetin) to apigenin strats with the hydrogenation of naringenin at C2-C3, catalyzed by flavone synthase. Subsequently, luteolin or diosmetin could be produced with the absence of Flavanoid3′-hydroxylase (F3′H) or Flavanoid3′5′-hydroxylase (F3′5′H), respectively (Pfeiffer and Hegedus 2011). Flavonols, such as kaempferol, quercetin, are synthesized from dihydroflavonols with the help of flavonol synthase. Under the catalysis of naringenin 3-dioxygenase, naringenin is hydroxylated at C3 to form the dihydrokaempferol. It can be converted further into dihydroquercetin or dihydromyricetin by F3′H or F3′5′H, respectively (Lucheta et al. 2007). For anthocyanin biosynthesis, dihydroflavonol reductase (DFR) catalyzes the reduction of dihydroflavonols into leucoanthocyanidins, which could be further reduced to anthocyanidins by anthocyanidin synthase (ANS) or catechin by leucoanthocyanidin reductase. Then anthocyanins could be glycosylated, acylated, or methylated at different sites, and transported by pecific transporters, such as glutathione S-transferase and so on (Tanaka et al. 2008).

In ornamental plants, transcriptional factors, such as MYBs, bHLHs, and WDRs, individually or coordinately regulated the expression of the flavonoid structural genes that determine flower colors (Naing and Kim 2018). The types of R2R3-MYB have been demonstrated to be involved in flavonoid metabolism and regulated the key genes of flavonoid biosynthesis (Albert et al. 2014; Xie et al. 2014; Zhang et al. 2017; Hichri et al. 2011). In cabbage, BoMYB1-3 take parts in anthocyanin biosynthesis. *BoMYB2* is more likely to be associated with anthocyanin production because it expressed more in red cultivars (Yuan et al. 2009). The MYBs would be suitable for metabolic genetic engineering for improvement of flower colors. The MYBs are vital candidates of metabolic genetic engineering for the improvement of ornamental crops.

It was reported that there are dynamic phytochemistry and metabolite changes of the common sunflower seed and sprouts (Guo et al. 2017). However, the roles of flavonoids metabolism in sunflower color development were unclear. In this study, we analyzed the transcriptomes and metabolomes in five ornamental sunflower cultivars. We found that more anthocyanins were accumulated in the red group flowers, especially the chrysanthemin. Some anthocyanins biosynthesis-related genes like UFGT (UDP-glycose flavonoid glycosyltransferase) also expressed more in the red group flowers. A MYB transcriptional factor, HanMYB1, was found to play vital positive roles in the modulation of anthocyanins metabolism by directly active the express of UFGT genes.

## Materials and methods

### Plant Materials

Five *Helianthus annuus* L. cultivars were planted in the field of Dongying, Shandong province, China. Each cultivar consisted of three biological replicates. The samples were collected at the flowering stage and then rapidly frozen in liquid nitrogen and stored at –80 °C.

### RNA-Seq analysis

Total RNA was extracted from *H. annuus*. L flowers, and the mRNA library of each sample was constructed and sequenced using Illumina HiSeq4000 (Illumina, San Diego, CA, USA) at Majorbio Bio-Pharm Technology Co., Ltd, Shanghai, China. In brief, the adaptor and low-quality sequences were removed using Fastp with default parameters(Chen et al. 2018), and clean reads were then mapped to the Sunflower genome (ftp://ftp.ensemblgenomes.org/pub/plants/release-49/fasta/helianthus_annuus) using HISAT2 (Kim et al. 2019), and integer read count calculate using the featureCounts function in the SUBREAD package in R (v1.2.5) (Liao et al. 2019). According to a previous method (Zhang et al. 2019), the DEGs analysis was conducted using classical normalization with the DEseq2 package in R(v1.2.5) with a 0.05 *p*-value and a four-fold-change cut-off limit (Love et al. 2014). All DEGs were analyzed by gene ontology (GO) enrichment using clusterProfiler and KEGG enrichment using eggnog-mapper tools (Yu et al. 2012; Huerta-Cepas et al. 2019). All raw data were uploaded in the Genome Sequence Archive at the Big Data Center, Beijing Institute of Genomics, Chinese Academy of Sciences, accession numbers CRA004924 (https://ngdc.cncb.ac.cn).

### Sample extraction and metabolomic analysis

Standard procedures as previously described by Chen et al. (Chen et al. 2014) were performed at Wuhan MetWare Biotechnology Co., Ltd (www.metware.cn). The freeze-dried sample was crushed using a mixer mill (MM 400, Retsch) with zirconia beads. 0.1g powders were weighed and extracted. Following centrifugation at 12,000 rpm for 10 min, the extracts were filtered before ultra-performance liquid chromatography tandem mass spectrometry analysis. Flavonoid extracts were analyzed using a liquid chromatography electrospray ionization tandem mass spectrometry system. Metabolite quantification was performed using a previously described scheduled multiple reaction monitoring method. Principal component analysis (PCA), hierarchical cluster analysis (HCA) and Kyoto Encyclopedia of Genes and Genomes (KEGG) pathway analysis were performed in the R software.

### Co-regulation cluster identification and analysis

We used Pearson’s correlation coefficient to construct metabolite regulatory networks based on the DEGs and different metabolite accumulation trends observed (Bishara and Hittner 2012). Mfuzz is a soft-clustering method based on the fuzzy c-means algorithm. (Kumar and Futschik 2007) Only genes with a correlation greater than plus or minus 0.85 were used for clustering.

### Subcellular localization assay

For the subcellular assay, the coding regions of HanMYB1 were ligated into pM999 and fused with the GFP. The construct was co-transformed with GHD7-RFP (a nuclear maker) into tobacco leaves, and the fluorescence signal was examined using a FluoView FV1000 confocal laser scanning microscope (OLYMPUS).

### Anthocyanin content assay

The Anthocyanin content was detected by Micro Plant Anthocyanin Assay Kit (Mibio). Briefly, 0.1 g samples were extracted in 1 mL extraction buffer and performed by ultrasound for 2 h. After centrifuging at 8000 g for 10 min, the supernatant was collected for further measurement. 20 μL supernatant and 180 μL buffer I were mixed for 20 min at 40 °C, then measured the absorbance at OD_530_ (A1) and OD_700_ (A2). Repeated the above procedure with buffer II, and obtained the A3 and A4. The content of Anthocyanin was calculated by the formula in manual.

### Dual-luciferase transient assay

To determine the transcriptional activation activity of HanMYB1, the reporter construct was produced by fusing a fragment of 2 kb of the promoter region of *UFGTs*, to the firefly luciferase gene. HanMYB1 were cloned into the effector vector to driven by the CaMV 35S. Renilla luciferase gene as an internal control. These constructs were contransformed into Arabidopsis protoplasts with a ratio of 6:6:1, and the luciferase activities were measured using the Dual Luciferase Reporter Assay System (Promega).

## Results

### Profiles of metabolome and transcriptome in different cultivars of sunflower

**Fig. 1:**
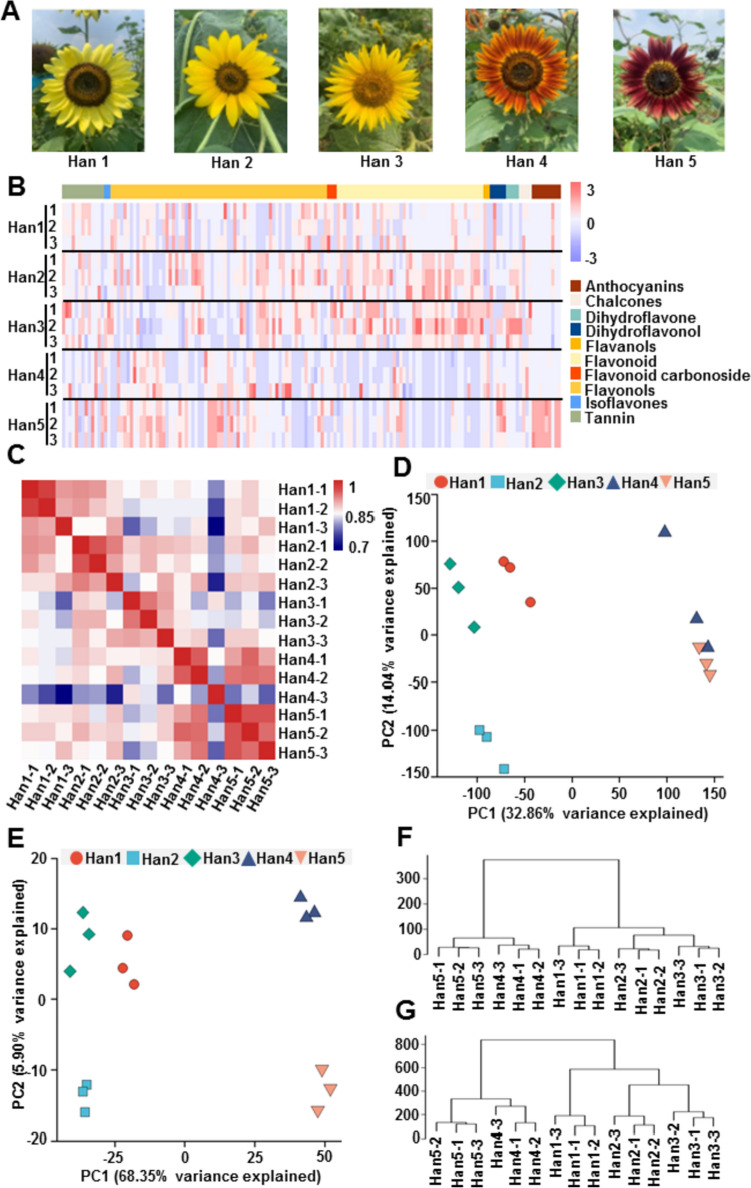
Metabolome and transcriptome analyses of *Helianthus annuus* L. cultivars with differently colored flowers. **A** Flower colors of the five cultivars in the flowering stage. **B** Overview of 154 annotated metabolites from five sunflower samples. Metabolites per row were *Z*-score standardized from −3 to 3. **C** Hierarchical clustering analysis of the expression profiles of 59,947 genes from samples of five sunflower cultivars. A color scale of 0–1 represents the Pearson correlation coefficient. **D** Principal component analysis of the metabolome data from five sunflower cultivars. **E** Principal component analysis of the transcriptome data from five sunflower cultivars. **F** Cluster dendrogram of the metabolome data of five sunflower cultivars. **G** Cluster dendrogram of transcriptome data from five sunflower cultivars (colour figure online)

Five ornamental sunflower (*H. annuus.* L) cultivars with lemon yellow (Han1), cream yellow (Han2), golden yellow (Han3), orange red (Han4), and rusty red (Han5) flowers were used in this study (Fig. 1A). They were different in flavonoid and anthocyanin content (Fig. S1). In total, 154 flavonoid metabolite species were identified, including nine anthocyanins, four chalcones, four dihydroflavones, five dihydroflavonols, two flavanols, 45 flavonoids, three flavonoid carbonosides, 67 flavonols, 2 isoflavones, and 13 tannins (Fig. 1B; Table S1). Hierarchical clustering analysis of gene expression profiles was performed on samples obtained from the five sunflower cultivars (Table S2). The results showed that the expression of genes was significantly different between the groups (Fig. 1C). Next, principal component analysis (PCA) of the metabolites and transcriptomes revealed ~32% and ~68% of variance among samples, respectively (Fig. 1D, E). The high similarity in the three biological replicates indicated that the analysis results were reliable and repeatable. PCA revealed clear clustering of the metabolome and transcriptome profiles according to flower color. It was obviously various in the differently colored samples, such as Han1 to Han3, and Han4 to Han5. A remarkably similar clustering result was observed in the dendrogram analysis for metabolome and transcriptome data from the five sunflower groups (Fig. 1F, G). Together the results indicate that flower color is modulated by differently accumulated metabolites and expressed genes.

### The analysis of the DAFs and DEGs in each cultivar

**Fig. 2:**
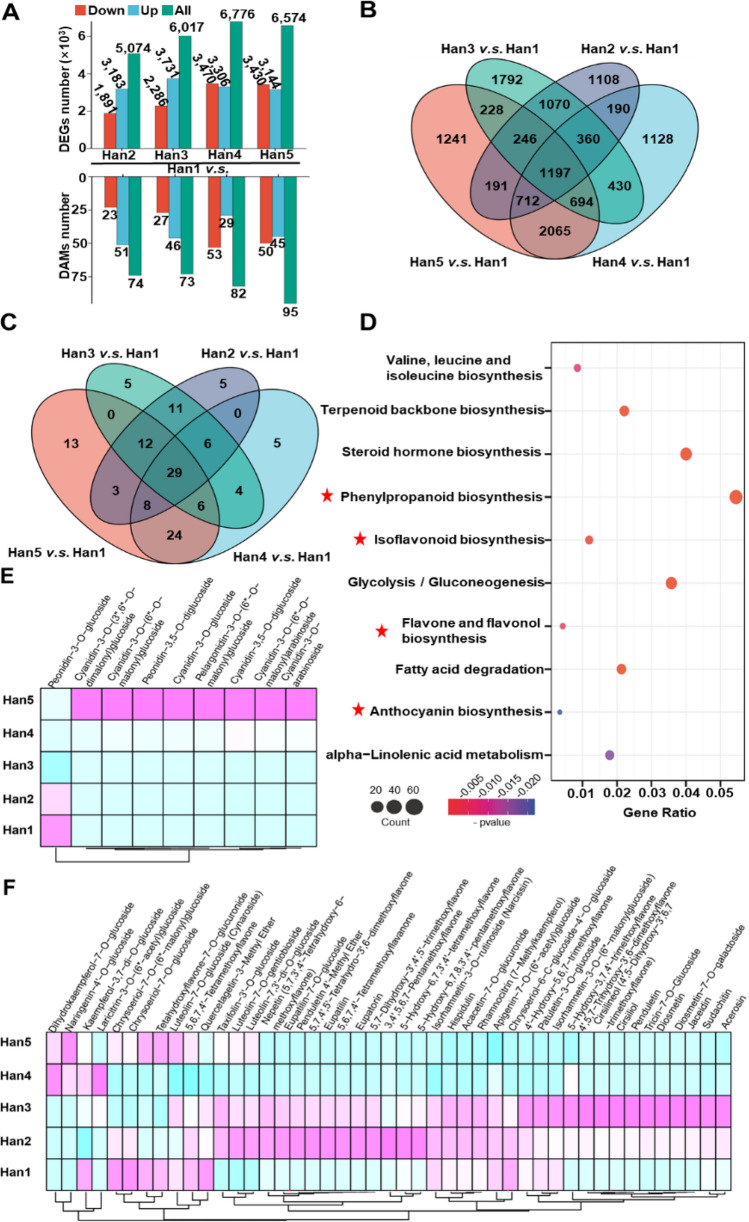
Identification and functional characterization of differentially expressed genes and differentially accumulated flavonoids (DAFs) in different samples. **A** Total numbers of DEGs (log_2_|FC| > 2 and *p* value <0.05) and DAFs (log_2_|FC| > 0.8, and VIP > 1) in each sample. **B** Venn diagrams depicting shared and distinct genes among four groups of samples. **C** Venn diagrams depicting shared and distinct metabolites among four groups of samples. **D** KEGG pathway enrichment analysis of DEGs in Han5 vs. Han1. The *y*-axis represents the pathway category and the *x*-axis represents the degree of enrichment of the pathway (gene ratio, number of DEGs in a given pathway divided by the total number of genes involved in the pathway). Circle color indicates the *p*-value, and circle size represent the number of genes involved. Significant enrichment was detected at *p* < 0.05. **E** Heatmap analysis of anthocyanin accumulation in five cultivars. **F** Heatmap analysis of flavonoid accumulation in five cultivars (colour figure online)

To insight into the potential roles of the flavonoid metabolites and associated genes in flower color formation, we analyzed the different accumulated flavonoid metabolites (DAFs) and differentially expressed genes (DEGs) in each cultivar (Han1 as the control, Fig. 2A–C; Tables S3, S4). The Venn diagram showed that both shared and individual-specific changes existed in flowers of different colors. Most specific DAFs and DEGs were founded in Han5, we tried to find the key elements by focusing on the DAFs and DEGs in Han5 vs. Han1.

The analysis of the DEGs was performed followed by Kyoto Encyclopedia of Genes and Genomes (KEGG) enrichment analysis (log_2_|FC|≥ 2 and *p*-value < 0.05). It was shown that the pathways for phenylpropanoid biosynthesis, isoflavonoid biosynthesis, flavone and flavonol biosynthesis, and anthocyanin biosynthesis were significantly different (Fig. 2D). Then, accumulation trends of anthocyanins and flavonoids in each cultivar are displayed in Fig. 2E and F. The results suggested that the Han1, Han2 and Han3 flowers accumulated relatively high amounts of flavonoids, and the Han5 flowers accumulated relatively high amounts of anthocyanins. The content of anthocyanins was detected and the results showed that Han5 and Han4 accumulated more anthocyanins (Fig. S1B). Overall, the results indicate that flavonoids and anthocyanins serve important functions in the development of sunflower colors.

### Flavonoid biosynthesis pathway and the color of *Helianthus annuus* flowers

**Fig. 3:**
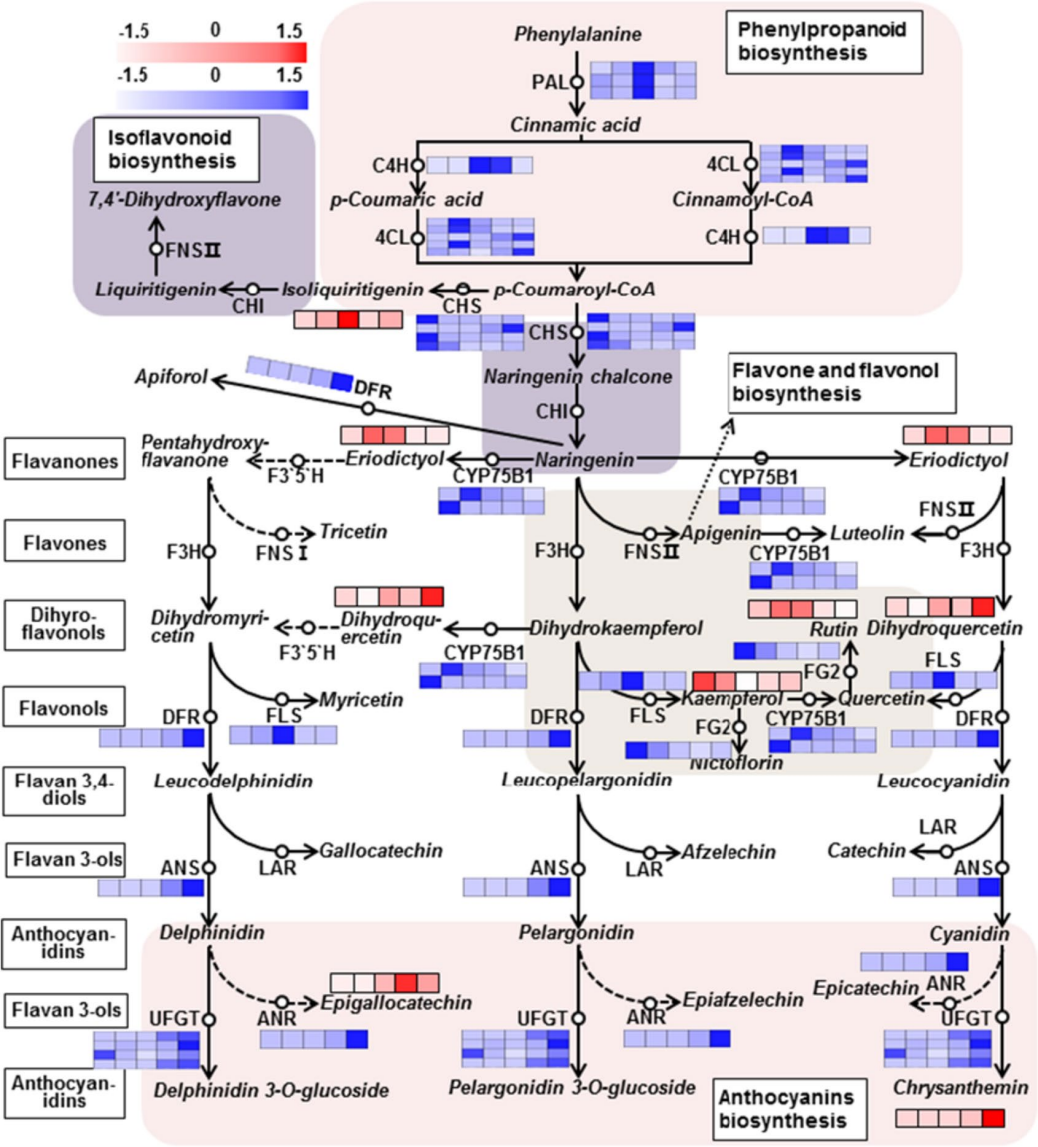
Flavonoid biosynthesis pathways in *Helianthus annuus* flower. Each colored cell represents the normalized intensity of each compound ion according to a color scale (three biological replicates for five cultivars, *n* = 15). Dotted arrows indicate the unidentified enzymes. The red bars denote different metabolite concentrations. Blue bar denotes relatively different gene expression levels, and from white to blue or red in the heatmap indicates the different expression levels of structural genes or metabolite contents ranging from low to high. Each cell from left to right represents Han1, Han2, Han3, Han4, and Han5. Enzymes in this pathway are as follows: PAL, phenylalanine ammonia-lyase; C4H, trans-cinnamate 4-monooxygenase; 4CL, 4-coumarate-CoA ligase; CHS, chalcone synthase; CHI, chalcone isomerase; CYP75B1, chalcone isomerase; F3H, naringenin 3-dioxygenase; DFR, bifunctional dihydroflavonol 4-reductase; ANS, anthocyanidin synthase; LAR, leucoanthocyanidin reductase; ANR, anthocyanidin reductase; FNSII, flavone synthase II; FLS, flavonol synthase; F3′5′H, flavonoid 3′,5′-hydroxylase; FNSI, flavone synthase I; UFGT, anthocyanidin 3-O-glucosyltransferase; FG2, flavonol-3-*O*-glucoside l-rhamnosyltransferase (colour figure online)

To explore the mechanisms of color development in *H. annuus* flowers, we further analyzed the flavonoid biosynthesis pathways of the five cultivars (Fig. 3). DEGs (denoted by blue bars) in the flavonoid biosynthesis pathway were identified, including phenylpropanoid biosynthesis, isoflavonoid biosynthesis, flavone/flavonol biosynthesis, and anthocyanin biosynthesis pathways. The DAFs are labeled in the metabolic pathway with a red bar. Twenty-four DEGs were enriched in the flavonoid biosynthesis pathway, including the *PAL, CHS, CHI, DFR, ANS,* and *UFGT*. Seven DAFs of the flavonoid biosynthesis pathway were detected, including isoliquiritigenin, eriodictyol, rutin, dihydroquercetin, kaempferol, chrysanthemin, and epigallocatechin. Anthocyanin (e.g., chrysanthemin and epigallocatechin) accumulation and the expression of key anthocyanin biosynthesis genes (ANS, ANR, DFR and UFGTs) were higher in red flowers (Han4 and Han5) than in the other flowers. Flavones or flavonols, such as rutin and kaempferol, were accumulated much more in yellow flowers, and the DEG trends exhibited similar patterns. The results revealed that chrysanthemin and epigallocatechin play vital roles in red sunflowers, and rutin and kaempferol play important roles in yellow sunflowers. In addition, the accumulation of the flavonoids was consistent with the key genes expressed in flavonoid biosynthesis pathways. Therefore, flavonoid biosynthesis plays a critical part in sunflower color development.

### MYB transcriptional factors and *Helianthus annuus* flower color

**Fig. 4:**
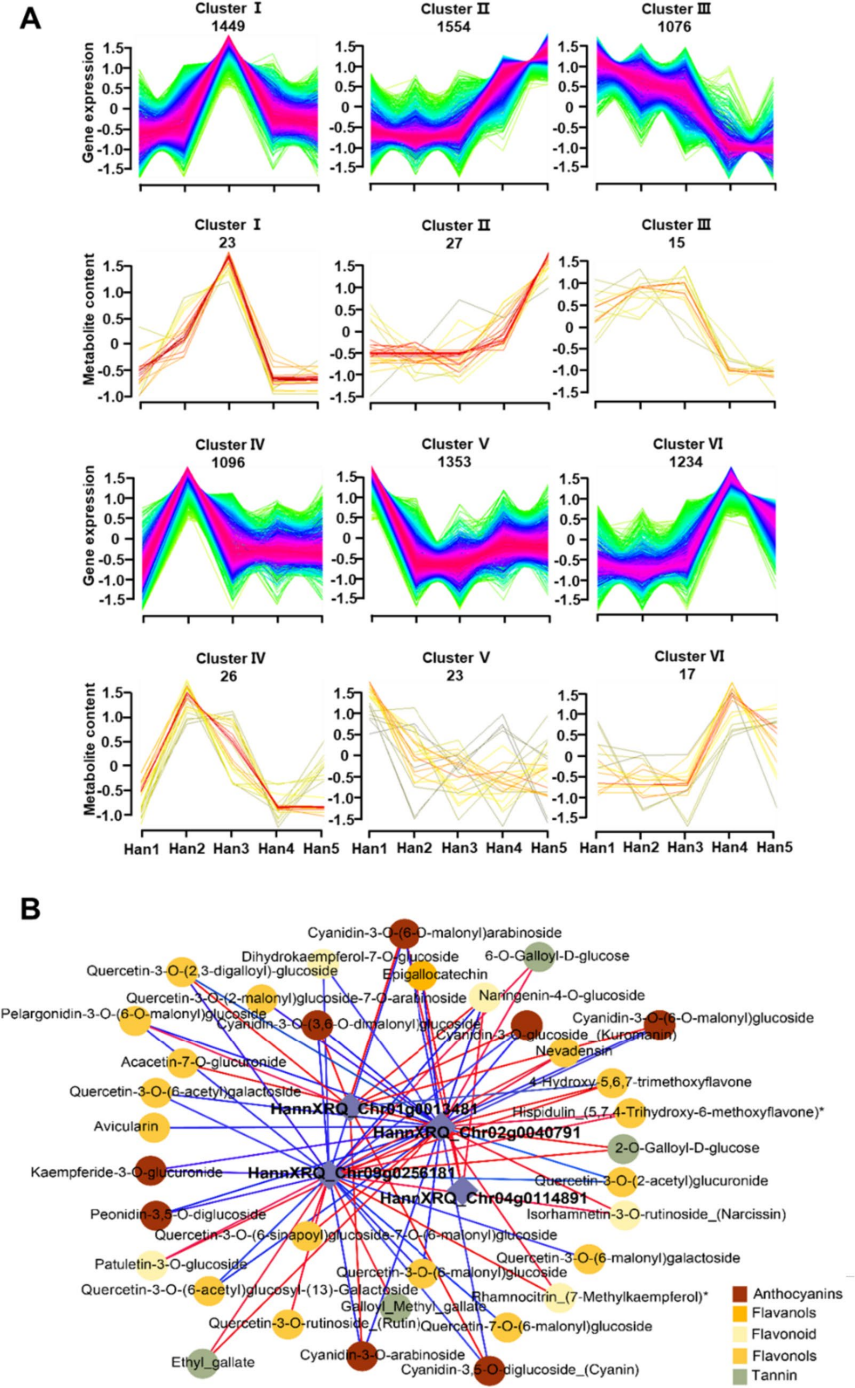
Analysis of co-expressed genes and metabolites. **A** Dynamics of metabolite accumulation and gene expression in the flower with different colors. The k-means clustering grouped the expression profiles of the sunflower metabolome and transcriptome into six clusters. The *x*-axis depicts five flower samples, and the *y*-axis depicts the standardized values of gene expression and metabolite content. The numbers shown in each box (for example, 23 metabolites and 1449 genes for cluster I) were derived from the number of metabolites and genes across all five samples in each cluster. **B** Correlation network constructed with MYBs (diamond) and differentially accumulated flavonoids (DAFs) (circle). Red line indicates positive correlation and blue line indicates negative correlation. |PCC| ≥ 0.85 and *p* value < 0.05 (colour figure online)

To obtain insights into the dynamics of gene expression and flavonoid accumulation trends in the different *H. annuus* cultivars, we performed a kinetic analysis using the transcriptomic and metabolome profiles in the five *H. annuus* cultivars. Six kinetic clusters of co-expressed genes and metabolites were obtained (Fig. 4A; Tables S5, S6). According to the expression or accumulation trends, clusters II and III were used to further studies. Cluster II encompassed 1554 DEGs and 27 DAFs, with increased levels in yellow to red color flowers, indicating that it was positively associated with the dark colors. Conversely, the DEGs and DAFs in Cluster III were negatively associated with red and dark coloration, as they were gradually downregulated.

In clusters II and III DEGs, some flavonoid-related transcriptional factors, such as MYBs and bZIPs, were observed among the DEGs. A network diagram was constructed using the PCCs (Pearson correlation coefficient) between the expression levels of transcriptional factors and the concentrations of 58 flavonoid metabolites (Fig. 4B; Table S7). The results showed that the four MYB transcriptional factors were the hub genes. The expression levels of HannXRQ_Chr02g0040791 and HannXRQ_Chr09g0256181 (cluster III members) were higher in the yellow group sunflowers (Fig. 4B; Fig. S2).The expression of HannXRQ_Chr01g0013481 and HannXRQ_Chr04g0114891 (clustered II member) were relatively high in red group sunflowers, and it was significantly and positively correlated with anthocyanin accumulation in the correlation network, especially HannXRQ_Chr01g0013481 (Figs. 4B; Fig. S2). These results indicated that HannXRQ_Chr01g0013481 may play vital role in flower color development.

### HaMYB1 modulates anthocyanin accumulation via *UFGT* genes

**Fig. 5:**
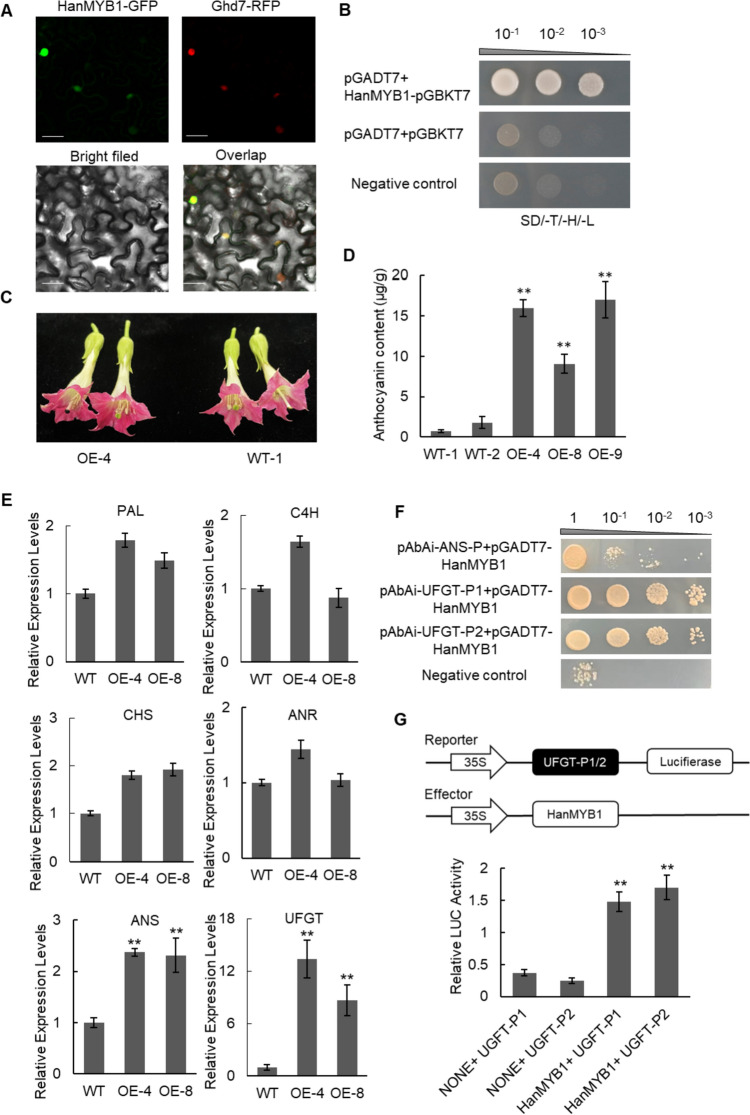
Characterization of HanMYB1. **A** Subcellular localization of HanMYB1. Ghd7-RFP is a nuclear marker. Bars = 25 μm. **B** Transcriptional activation activity of HanMYB1 in yeast. **C** The flower color of *HanMYB1* overexpression lines. **D** Anthocyanin accumulated more in *HanMYB1* tobacco overexpression lines. Error bars indicated the standard deviations based on three replicates (** *p* < 0.01, Student’s *t* test). **E** Expression levels of anthocyanin biosynthesis-related genes in *HanMYB1* tobacco overexpression lines. Error bars indicated the standard deviations based on three replicates (** *p* < 0.01, Student’s *t* test). **F** HanMYB1 bound to the promotor of *UFGT*s by Y1H assay. The selective medium containing 20 ng/mL AbA. **G** Effect of HanMYB1 on the promotors of *UFGTs* using a dual-luciferase transient assay in Arabidopsis protoplasts. Error bars indicated the standard deviations based on three replicates (** *p* < 0.01, Student’s *t* test) (colour figure online)

According to the sequence homology, HannXRQ_Chr01g0013481 could be identified as a MYB transcriptional factor because of containing Myb/SANT-like domain (Fig. S3A). It was named as *HaMYB1* based on its location on chromosome (Fig. S3B). To understand the subcellular localization of HaMYB1, it was fused to GFP protein and co-transformed with nuclear protein GHD7-RFP into tobacco leaves. The results of overlapped fluorescence showed that HaMYB1 was a nuclear protein (Fig. 5A). The yeast transactivation assay results showed that HaMYB1 has the transcriptional activation activity (Fig. 5B).

For investigating the possible roles of HaMYB1 in anthocyanin accumulation, we generated transgenic tobacco overexpressing *HaMYB1* and tested these plants for anthocyanin content (Fig. S3C; Fig. 5B, C). The flower color was deeper in *HaMYB1* overexpression lines (Fig. 5C). And the flower of the overexpress lines accumulated more anthocyanin than the negative control transgenic lines (Fig. 5D). Then the expression levels of anthocyanin biosynthesis-related genes were further investigated in transgenic lines by RT-qPCR. The expression levels of *ANS* and *UFGT* were significantly increased in the *HanMYB1* overexpression lines (Fig. 5E). Considering the different trasnscriptional levels of *ANS* and *UFGT* in the various *H. annuus* cultivars (Fig. 3), we proposed that *ANS* and *UFGT* may be regulated by HanMYB1. Fine analysis of the promotor sequences revealed that remarkable motifs in the promotor of *ANS* (HannXRQ_Chr04g0095471) and *UFGT* (UFGT-P1, HannXRQ_Chr08g0231931; UFGT-P2, HannXRQ_Chr10g0288021). The yeast one hybrid assay showed that HanMYB1 could bind to the promotors of *UFGTs*, rather than ANS (Fig. 5F). Then HanMYB1 was fused to CaMV-35S promotor as effector and the promotors of *UFGTs* were fused to CaMV-35S promotor and firefly luciferase as reporter. These vectors were co-transfected into the Arabidopsis protoplasts (Fig. 5G). Simultaneously, the Renilla luciferase gene driven by the 35S promoter was co-transfected as an internal control. The data indicated that HanMYB1 could obviously activate the expression of *UFGT* genes. Taken together, these results suggested that HanMYB1 functions as positive regulator in anthocyanin accumulating.

## Discussion

In long-term cultivation and domestication, sunflower has acquired great diversity, especially in terms of flower color, which greatly enhances its ornamental value (Cvejic et al. 2020). Flavonoids are one of the major pigments in higher plants, and flavonoid types, such as anthocyanins, chalcones, aurones, and some flavonols, are the major flower pigments (Iwashina 2015). In the present study, five ornamental *H. annuus*. L cultivars, with flower colors ranging from Han1 to Han5 were used to study the mechanisms of sunflower color formation (Fig. 1A). According to the transcriptomic and metabolomic analyses, we revealed that their DEGs and DAFs were highly associated with flower color (Fig. 1B–G). The yellow group (Han1–Han3) and the red group (Han4 Han5) were clearly separated based on DEGs and DAFs. Notably, the Han4 and Han5 flowers had high numbers of DAGs and DAFs, in comparation with the Han2 and Han3 flowers (Fig. 2A). The results indicated that the different flavonoid accumulation and gene expression trends were responsible for the differently colored flowers in the *H. annus* cultivars studied.

The red colors observed in seed-cone bracts of some living conifers are reportedly linked to the accumulation of anthocyanin pigments (Rudall 2020). The yellow flowers of *Gossypium spp.* (Malvaceae) and *Tagetes spp.* (Asteraceae) accumulate yellow flavonols such as gossypetin, quercetagetin, and kaempferol and quercetin glycosides. In our study, the red group flowers accumulated more anthocyanins and the yellow group flowers accumulated more flavonoids (Fig. 2E, F). Higher levels of chrysanthemin and epigallocatechin were detected in the red-colored sunflowers, and higher levels of rutin and kaempferol were accumulated in the yellow-colored sunflowers (Fig. 3). Besides, the dynamic transcriptional level of flavonoid biosynthesis associated genes accompanied with the accumulation of some metabolites. Four MYB transcriptional factors may play vital roles in the regulation of flavonoid accumulation (Fig. 4B). Here, we reported that HanMYB1 (HannXRQ_Chr01g0013481) play a positive role in the regulation of anthocyanin accumulation by directly promoting the *UFGT* genes (Fig. 5). Coincidentally, *HanMYB1* was located on LG1 (2.25 cM) which is said to be significantly associated with the presence of anthocyanins in cultivated sunflower, with an effect size of 7.5% and increased the likelihood of presence by 12% (Dowell et al. 2019). Above all, these findings provide a sound theoretical basis for the genetic improvement of ornamental sunflowers.

### Supplementary Information

Below is the link to the electronic supplementary material.

Figure S1. Flavonoid and anthocyanin contents in the five cultivars. (PNG 20 KB)

FigureS2. The expression levels of the four MYBs in the five cultivars. (TIF 348 KB)

Figure S3. Identification of HanMYB1. Sequence analysis of HannXRQ_Chr01g0013481. The phylogenetic relationship of MYB transcriptional factors in sunflowers and Arabidopsis. The expression levels of HanMYB1 in overexpression lines. (XLSX 43 KB)

Supplementary file4 (XLSX 14798 KB)

Supplementary file5 (XLSX 4924 KB)

Supplementary file6 (XLSX 64 KB)

Supplementary file7 (XLSX 2073 KB)

Supplementary file8 (XLSX 38 KB)

Supplementary file9 (XLSX 13 KB)

Supplementary file10 (PNG 12 KB)

## Data Availability

All raw data were uploaded in the Genome Sequence Archive at the Big Data Center, Beijing Institute of Genomics, Chinese Academy of Sciences, accession numbers CRA004924 (https://ngdc.cncb.ac.cn).
