# Supplementary figures and images for: Integrated transcriptome and metabolome analysis revealed that HaMYB1 modulates anthocyanin accumulation to deepen sunflower flower color
Source: Plant Cell Rep. 2024 Feb 21;43(3):74. doi: 10.1007/s00299-023-03098-3 (PMC10879246; doi:10.1007/s00299-023-03098-3)

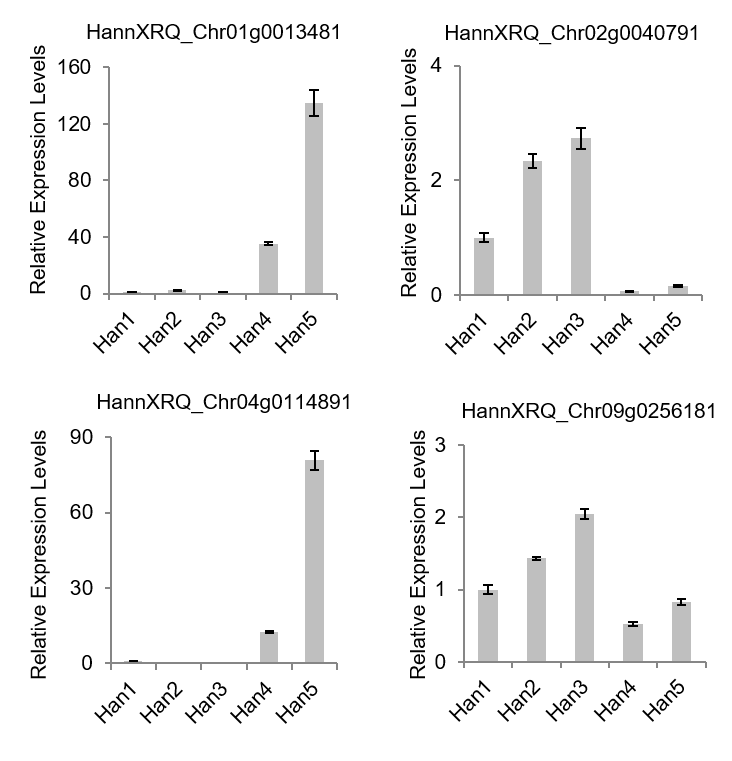

Supplement: Supplementary file 1 — Figure S1. Flavonoid and anthocyanin contents in the five cultivars. (PNG 20 KB) [file 299_2023_3098_MOESM1_ESM.png]

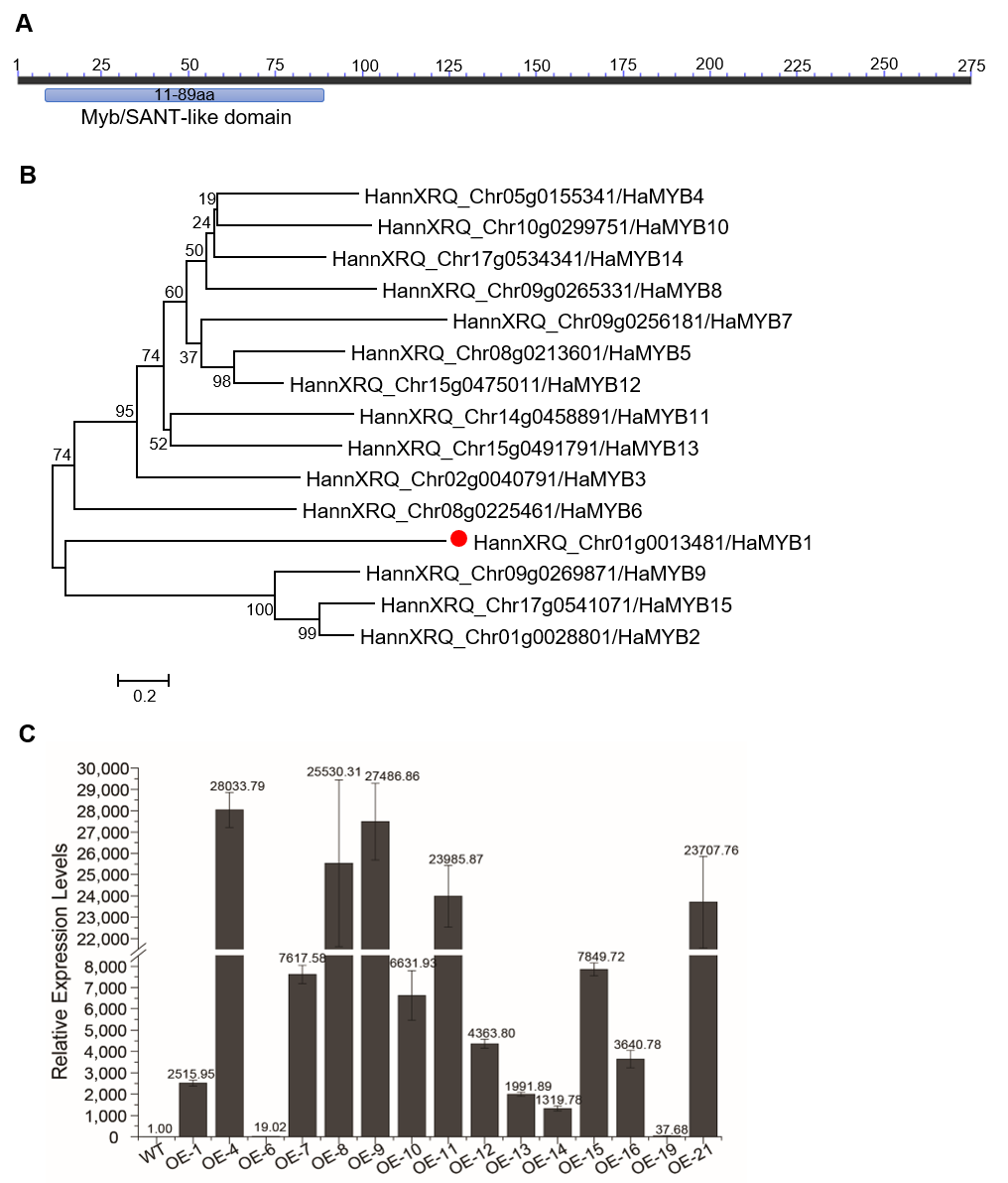

Supplement: Supplementary file 2 — FigureS2. The expression levels of the four MYBs in the five cultivars. (TIF 348 KB) [file 299_2023_3098_MOESM2_ESM.tif]

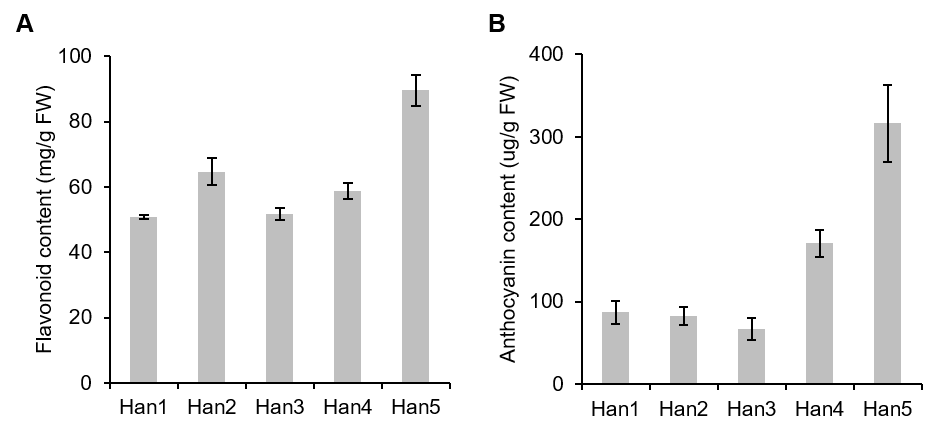

Supplement: Supplementary file 10 — Supplementary file10 (PNG 12 KB) [file 299_2023_3098_MOESM10_ESM.png]
